# Using Photovoice to Improve Engagement in Community Health Assessments Addressing Behavioral Health

**DOI:** 10.1007/s11414-024-09885-4

**Published:** 2024-05-06

**Authors:** Stacey Li, Jennifer Gulley, Marisa Booty, Bradley Firchow, Margaret L. McGladrey

**Affiliations:** 1 School of Public Health and Tropical Medicine, Tulane University, New Orleans, LA 70118 USA; 2 Clark County Health Department, Winchester, KY 40391 USA; 3 University of Kentucky College of Arts and Sciences, Lexington, KY 40506 USA; 4 University of Kentucky College of Medicine, Lexington, KY 40506 USA; 5 University of Kentucky College of Public Health, Lexington, KY 40536 USA

## Abstract

Behavioral health disorders are well-known to have close links with the social determinants of health, yet little is known about how impacted communities perceive these links. Qualitative participatory methods can not only provide insight into how communities conceptualize these relationships but also empower those with lived experience to contextualize their perspectives and formulate calls to action. This study used Photovoice as a participatory method to supplement the Clark County Health Department Community Health Assessment and determine priority facilitators and barriers contributing to the behavioral health of Clark County, KY, residents. A secondary aim was to gain a greater understanding of how the Photovoice methodology impacts community engagement efforts in Community Health Assessments. Twenty-three Clark County residents participated in four Photovoice groups involving five weekly sessions, which included photograph “show and tell,” critical group dialogue, participatory analysis, and planning for dissemination. Secondary analysis of Photovoice focus group discussions revealed behavioral health facilitators and barriers were most influenced by (1) public sector unresponsiveness, (2) strong partnerships formed between community and grassroots organizations, and (3) the siloed division of responsibility between agencies and across sectors. The authors also found the Photovoice method successfully enhanced engagement and empowered those with lived experience to frame their perspectives of the behavioral health landscape. This project has implications for enhancing community engagement and empowerment in behavioral health–focused public health assessments and shaping policy to promote multi-sector collaboration.

## Introduction

### Community-Based Assessments of the Social Determinants of Behavioral Health

Behavioral health issues (e.g., mental illness, substance use disorders [SUDs]) remain a major public health crisis in the United States. The National Survey on Drug Use and Health estimated that 59.4 million American adults had a mental illness in 2022, and 13.2 million adults had thoughts of suicide.^2^ Additionally, 17.3% of those 12 and older had a SUD, although 94.7% of adults did not receive treatment.^2^ These data underscore the pressing need to improve safety nets given the impact of social determinants on behavioral health outcomes.^3^ The biopsychosocial model acknowledges that SUDs and mental illnesses are biological conditions interwoven with social, community, and environmental factors, including employment, healthcare access, urbanicity, and poverty^3–10^ It is also important to recognize the two-way connection between behavioral health and social determinants, as behavioral health can affect social issues including homelessness, legal system involvement, education, and economic insecurity.^3,11–13^ Given this complex interplay, it can be difficult to prioritize the specific health or social determinants in communities that require immediate focus and resource investment to improve behavioral health outcomes.

To identify these strategic priorities, many local health departments conduct Community Health Assessments (CHA) and subsequent Community Health Improvement Plans (CHIP) every 5 years to achieve and maintain accredited health department status by the Public Health Accreditation Board (PHAB).^14^ Some unaccredited health departments also choose to complete a CHA/CHIP considering the evidence that routine utilization improves public health decision-making and actions.^15^ Numerous guides have been published on best practices for CHA/CHIP processes, with broad, proactive, and diverse community engagement designated as a foundational component.^16–22^ Such collaborative action is particularly crucial in addressing the behavioral health crisis by elevating the voices of those marginalized by stigma or discrimination.^23,24^ Community-engaged needs assessments also combine a range of perspectives for collective questioning and planning, which ultimately allows for knowledge production rather than solely data collection on what is already known.^25–27^

One established approach to community-engaged needs assessments is Photovoice, a powerful qualitative participatory action research method that has been used to study SUDs and mental illness, including designing recovery interventions,^28^ eliciting insights about lived experiences,^29–32^ illustrating protective factors for recovery in minoritized populations,^33^ and combatting stigma.^34,35^ For example, one study demonstrated the use of Photovoice among minoritized SUD clients to drive discussions regarding policy change, generate knowledge on recovery, and improve culturally appropriate services.^33^ A recent review described Photovoice as an “ideal method for research” for those in recovery because its participant-driven nature allows for a more complex understanding of individual narratives.^36^ The method involves engaging community stakeholders to identify, represent, and enhance their community through photography and critically analyze illustrated issues in group dialogue.^37^ To achieve these goals, Photovoice projects entail the following: (1) organizing participants as co-researchers to identify and photograph facilitators and barriers to community wellbeing, (2) guiding critical group dialogue with the SHOWeD method to analyze the identified facilitators and barriers,^38^ and (3) reaching decision-makers to advocate for change in response to identified community needs.^37,39–41^

The participatory nature of Photovoice makes this methodology particularly appropriate to inform CHA/CHIP efforts by synergizing their goals of determining, recording, and reflecting on community facilitators and barriers, addressing issues, and promoting stakeholder ownership with community-driven dialogue of change.^37^ Additionally, the potential of Photovoice to increase engagement with and empowerment of stigmatized communities is especially important for behavioral health–focused public health assessments.^42^ Unfortunately, reporting on Photovoice projects typically omits evaluation of the characteristics of decision-making audiences of Photovoice exhibits, the effects of the experience on participants and other community stakeholders, and the outcomes of exhibitions on policy-making and social change, which is important to assess given that the third facet of Photovoice defined by the method’s originators emphasizes having actionable impacts on policy.^37,39,40,43,44^ Embedding Photovoice into CHA/CHIP strategic planning and local policy-making can enhance the evaluation of its instigated social and policy change through the CHA/CHIP’s cyclical monitoring structure.

### Community-Engaged Behavioral Health Assessment Using Photovoice: A Kentucky case

Although the importance of community ownership of CHA initiatives is well-known, successful implementation of robust engagement remains a challenge.^45,46^ Previous research utilizing community participation focused on interventions rather than identifying health facilitators or barriers,^47^ have not cited participation frameworks or emphasized power structures,^47–49^ and have tended to focus heavily on the perspectives of healthcare leaders and practitioners rather than those of affected individuals and communities.^50–52^ Despite the potential for CHAs, Photovoice, and behavioral health research to enhance one another, there are currently no studies that combine their goals.

This study addresses these research gaps by leveraging the local knowledge of community members in rural Clark County, KY, to collaborate with the Clark County Health Department (CCHD) through their 2022 CHA, with many participants contributing their expertise of lived behavioral health experience. Rural Clark County is a prime context in which to study efforts to improve social determinants of behavioral health considering the extent of the mental health and SUD crisis. In comparison to the United States population, Clark County residents have disproportionately higher rates of overdose deaths per 100,000 (65 vs. 23 in Clark County vs. US respectively), poor mental health days per 30 days (6.0 vs. 4.4), frequent mental distress (18% vs. 14%), patient to mental health provider ratios (1000:1 vs 340:1), and adult smoking (23% vs. 16%), which are heightened by social disparities such as higher prevalence of poverty status (15.4% vs. 11.5%), food insecurity (14% vs. 12%), and lower access to exercise opportunities (77% vs. 84%).^53–55^ In a 2022 survey of 46 local health departments across Kentucky, substance use (73.9%) and mental health (52.2%) were the top two most commonly identified CHIP focus areas.^56^ Given the severity of this public health issue, this case study aims to uncover the perceived facilitators and barriers to behavioral health using Photovoice in a rural Kentucky county. A secondary goal is to gain a greater understanding of employing this method to improve community engagement in CHA initiatives.

## Methods

### Primary Data Collection

The current study began after the CCHD Director of Performance Management and Accreditation (and second author) experienced the Photovoice method as a participant in the HEALing Communities Study, an active randomized waitlist controlled trial funded by the National Institute on Drug Abuse and Substance Abuse and Mental Health Services Administration.^57–59^ In late summer 2022, the second author reached out to the senior author to implement the Photovoice method in the CCHD CHA process. In September 2022, the CCHD recruited 23 Clark County residents through listservs, civic groups, coalition meetings, pop-up events, and other communication channels. The study received a Not Human Subjects Determination from the Institutional Review Board at the senior author’s institution as it was classified as a quality improvement project and thus exempt from further oversight. Researchers created an abbreviated consent form (not IRB-reviewed) to inform participants about data usage for the CHA/CHIP process.

#### Procedures

**Figure 1:**
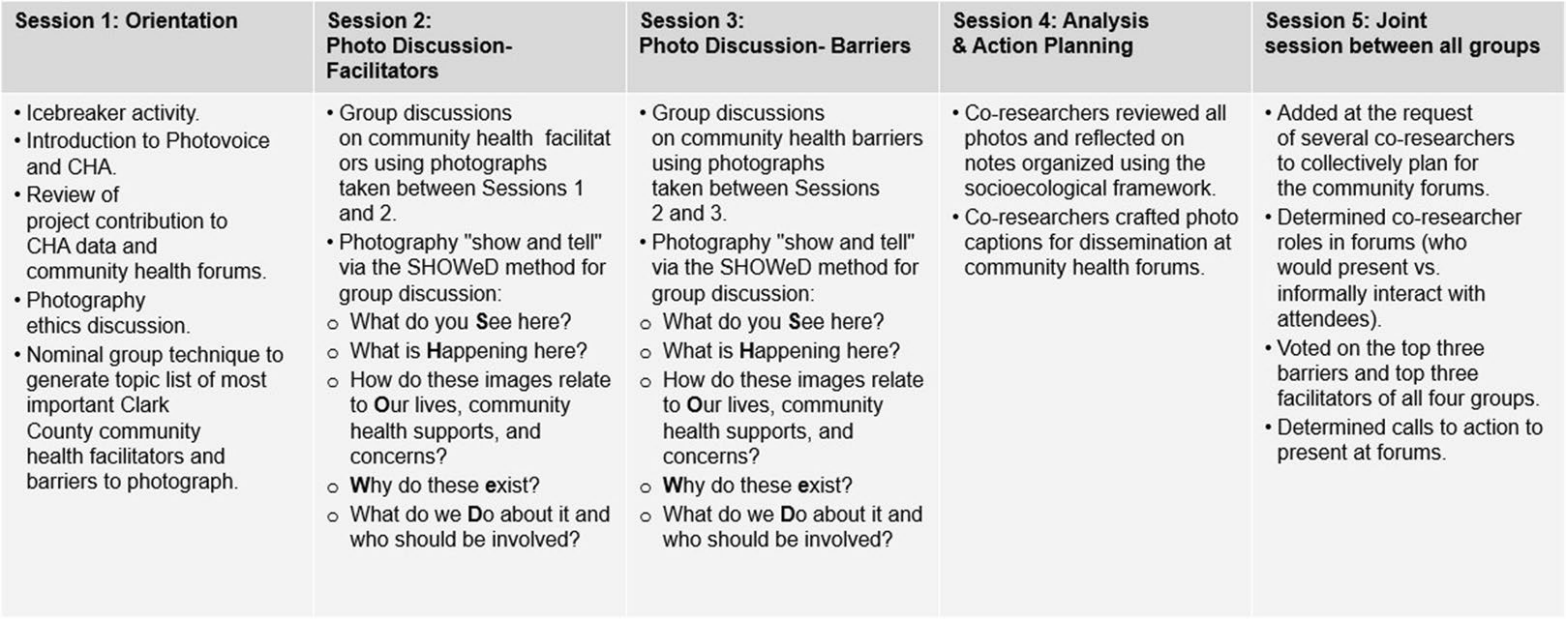
Procedures of each session

The second author organized four weekly Photovoice groups in October and November 2022 with the third author (note-taker/analyst) and senior author (primary coordinator). Participating co-researchers were assigned to their group based on scheduling availability (e.g., preference to meet on Tuesday evenings vs. Wednesday mornings) and received a $10 gift card for each session. The 90-min sessions, with participant permission, were audio-recorded, transcribed verbatim using Zoom’s business account feature (names replaced by ID numbers), and reviewed for typographical errors by the third author. Sessions included an orientation using the nominal group technique to generate a list of community health facilitators and barriers to photograph in Clark County.^60^ This was followed by two photo discussion sessions and an analysis and action planning session for co-researchers to collaboratively analyze their photos, notes, and prior discussions for dissemination at community health forums. Discussions followed the framework of the SHOWeD method, which includes a “show and tell” of photos with co-researchers sharing what their images picture, where they were taken, how they connect to community health facilitators or barriers, discussions of prevalence, why the issues or assets exist, what to do about these issues, and key players who should be involved with solutions.^38^ A fifth joint session was added at the request of some co-researchers to collectively plan for the community forums. Specific details on each session and the SHOWeD method are included in Figure 1. Procedures followed established methods for conducting and reporting qualitative research, as per the Standards for Reporting Qualitative Research.^1^

### Participatory Analysis

Coordinators took notes and analyzed discussions during Sessions 2 and 3 employing the Socioecological Framework,^61^ identified key themes, and presented them to co-researchers for reflection in Sessions 3 and 4. In Sessions 4 and 5, co-researchers analyzed facilitators and barriers with organized notes, quotes, and photos. Participants used the analysis and Photovoice insights to choose each group’s top three community health facilitators and barriers, write captions, and prioritize “calls to action” for the CHA effort. The coordinators and a contracted graphic designer (also a Photovoice group member) created displays of the top-three rankings and participant-generated photo/caption combinations in the form of a presentation, posters, and postcards for community health forums in November and December 2022.

### Community Forum Exhibit and Evaluation

Co-researchers submitted 112 photos and developed 89 photo/caption combinations. For the community forums and subsequent coalition meetings, 50 postcards were printed of each photo/caption combination for distribution. A poster was produced for each group’s top three facilitators and top three barriers as well as a larger movie-sized poster reflecting the top three facilitators and barriers of all groups combined. The final poster and postcards are available at https://www.ccactivitycoalition.com/photovoice2022.^62^ Each group displayed their poster and postcards at two community forums with group representatives on hand to answer questions. Between the forums, the county judge executive placed the Photovoice posters in the courthouse to increase awareness and to promote the December forum.

Online and paper surveys were distributed to collect feedback from Photovoice co-researchers and community forum attendees, assessing project goals achievement and the impact of the Photovoice process on the forums. Using Likert-type scales, participants were asked to indicate the extent to which they felt involved and empowered by their Photovoice work and the extent to which the project met Photovoice’s three goals. The survey included five open-ended questions for participants to describe their Photovoice experience, the most impactful elements, and the intended actions inspired by the project.

### Secondary Thematic Analysis

The first author conducted a secondary thematic analysis on data from the participatory analysis, including photo/caption combinations, topically organized notes, transcripts, and evaluation data, to identify underlying themes of participant-generated facilitators and barriers to community behavioral health. Consistent with participatory action research principles, photos were coded based on the content of associated captions that participants wrote. NVivo software was used to develop themes with Braun and Clarke’s six-phase qualitative thematic analysis method.^63^ This process involves familiarizing analysts with the data, generating initial codes, developing themes, reviewing potential themes, defining themes, and producing the report.^63^ Given the exploratory nature of the study, predominantly inductive analysis was initially employed to create behavioral health-specific topical codes using participant-identified topics as a starting framework (e.g., access to behavioral healthcare, harm reduction, stigma). Broader categories and preliminary themes were incorporated as additional latent codes to identify underlying systemic successes or failures that shaped behavioral health (e.g., awareness, grassroots efforts, community partnerships). Considering the broad range of determinants, the authors felt it important to incorporate data from discussions that were not explicitly about SUD or mental illness but still potentially impacted behavioral health in larger structural or systemic ways. After applying the manifest behavioral health topic codes to analyze data that explicitly addressed behavioral health, the first author completed a deductive analysis using behavioral health a priori codes on discussions that did not explicitly mention behavioral health. The first and senior authors met weekly–biweekly to reflect on the coding process, determine links between major concepts, and review developing themes. A final member-check exercise reviewed the results of the secondary analysis with participants to incorporate feedback during the manuscript revision.

## Results

### Sample Characteristics

Twenty-three Clark County co-researchers, aged 26 to 73 (mean 49 years), joined the Photovoice team. Participants, diverse in education, included a higher proportion of non-white and female members compared to the general Clark County population.^54^ As of July 2022, Clark County had a population of 37,061 and is classified as rural by its Rural–Urban Commuting Area code using the 2020 census population data.^54,64^ Both community forums had a combined attendance of 76 individuals and representation from 24 organizations. See Table 1 for additional characteristics.

**Table 1:** Sample characteristics

Characteristic	Photovoice co-researcher (*n* = 23)	Forum audience member (*n* = 76)
Age, years, %		
18–30	1.3	9.3
31–45	48.6	37.4
46–60	15.6	32.0
61–75	34.5	19.0
76 +	0.0	1.3
Sex, %		
Male	30.5	29.3
Female	69.5	70.7
Race/ethnicity, %		
White	82.6	91.9
Black	17.3	5.3
Asian	4.3	0.0
Hispanic	4.3	0.0
Other or N/A	0.0	2.8
Education, %		
High school degree or equivalent	22.0	15.7
Some college, no degree	22.0	17.0
Associate degree	8.7	7.2
Bachelor’s degree	30.4	35.6
Graduate education	13.0	21.2

### Facilitators and Barriers to Community Behavioral Health

Although not explicitly asked, co-researchers discussed behavioral health extensively due to its interconnectedness with other community health topics. A participant highlighted behavioral health as a public health crisis, stating, “This [behavioral health challenges] is a big problem in Clark County and a lot of counties in Kentucky and over the nation.” During the generation of community health topics, behavioral health was consistently mentioned in all four groups, with the following identified as crucial for depiction through photos: mental health and recovery stigma, lack of mental healthcare providers and services, changes from punishment to treatment, harm reduction, resources for dual diagnoses, SUD support groups, overdose emergency response, connections between SUD and housing, need for mental health awareness, problems of recidivism for those with mental illness, lack of employment and education opportunities for those in recovery, need for more rehab and sober living facilities, and SUD crises in youth. Thematic analysis revealed the barriers and facilitators to community behavioral health fell within three major themes: (1) individuals vs. government: who’s responsible?, (2) the power of partnerships among community organizations, and (3) linking facilitators and barriers: the cycle of public unresponsiveness to local calls for behavioral health investments.

#### Barriers: Individuals vs. Government: Who’s Responsible?

**Figure 2:**
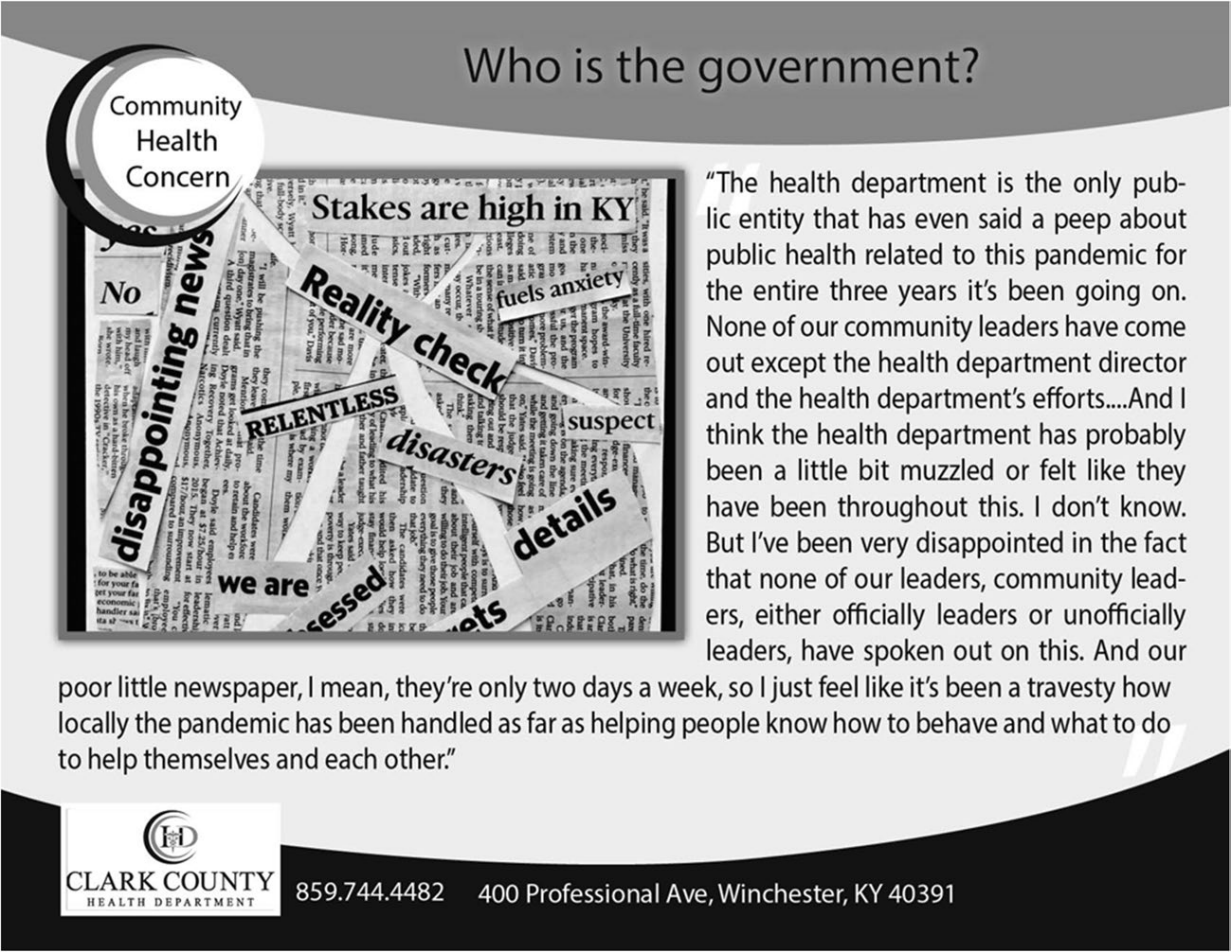
Photo and caption on postcard illustrating community health barrier

Co-researchers identified the following as the most pressing community health barriers: environmental justice and hazardous conditions, lack of affordable housing and public infrastructure, local government non-responsiveness to community health issues, health disparities between “haves and have-nots,” lack of safe transportation or walkability, loss of community activities/shopping venues/restaurants, and intersections of houselessness with behavioral health challenges. Conversations highlighted the insufficiency of public sector systems as an underlying contributor to these community health barriers. The following quote underscores the lack of government responsiveness as a major barrier to overall community health: “I would like to say we need to involve our county officials and stuff in this. But that’s not going to work… It’s not something the mayor’s gonna go help.” Another participant ironically stated, “They’re [the government] supposedly in charge of keeping citizens safe.” An additional concern of government responsiveness is illustrated in Figure 2.

Co-researchers echoed this frustration with public sector support for behavioral health. A participant who was an employee of a recovery nonprofit explained, “The expectation was for the (nonprofit) director to present to city commission and use our own funds that we raise, not taxpayer funds, to do projects. And it was really hard to get anything to take one step forward.” In the absence of government-provided upstream safety nets, downstream services, and funding, passionate “boots on the ground” individuals are left to “do it ourselves” to implement solutions without structural supports. In the words of one co-researcher, “Citizens don’t wait for the government to do things. They get things done, they do it themselves.” Co-researchers perceived the work of caring individuals and community groups as the sole reliable solution to behavioral health issues in the absence of public sector support. Emphasis was placed on grassroots and volunteer efforts in the non-profit sector carrying a significant portion of responsibility. One co-researcher explained, “Most agencies that serve people in this community have been a grassroots venture that have grown out of an identified problem.” Volunteers and non-profit employees expressed the need to “step up” and bridge gaps in the fragmented behavioral healthcare continuum. One co-researcher described their community dynamics as “good people giving back, regardless of not being heard by their elected officials.” Frustration arose regarding the responsibility falling on grassroots initiatives led by service users, passionate family members, and community allies to navigate challenges and implement solutions.

#### Facilitators: The Power of Partnerships Among Community Organizations

Participants identified the following as key community health facilitators in Clark County: community gatherings, assets in the social safety net, services and supports promoting community health, access to health/community health assets, parks, and recreation, Clark Regional Medical Center expansion and leadership, and resources working together to meet community needs. Community partnerships were central to many facilitators and significantly contributed to their inherent strengths. A co-researcher stated, “They have all just morphed out of folks discussing together community problems and then bringing in other people to try to help solve them. And the strength of a collaborative community, it’s been a major factor in things happening here.” Many behavioral health and related social service organizations were recognized as significant community supports: “It seems like Clark County has a good amount of private nonprofit organizations that serve health needs. That’s definitely a benefit.” Co-researchers highlighted partnerships among non-profits and coalitions facilitating behavioral health services; for example, one said, “Without everybody, this place wouldn’t stand a chance. You need all those partners.” Another corroborated this by saying, “All the community partners, without the community, we can’t do what we do.”

**Figure 3:**
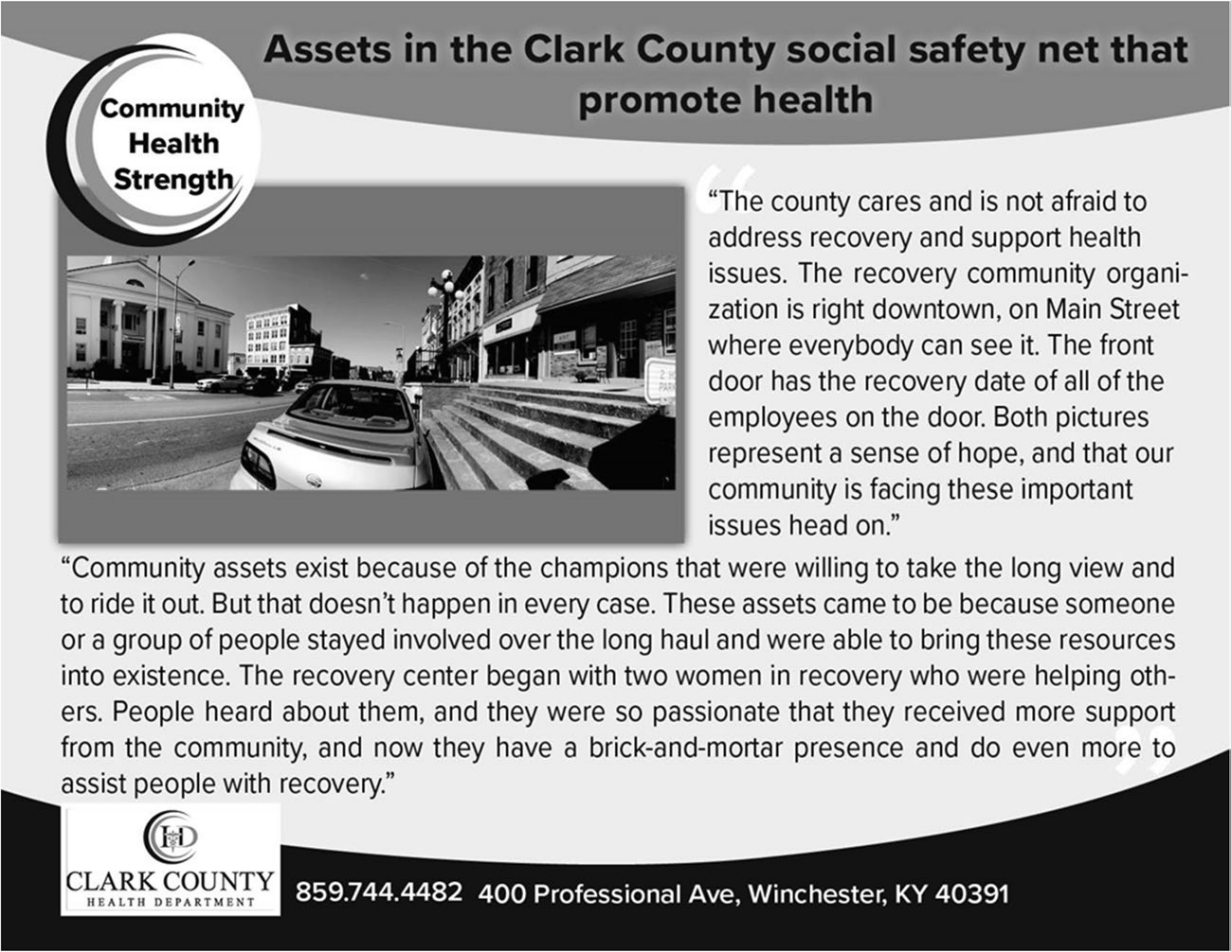
Photo and caption on postcard illustrating community health facilitator

**Figure 4:**
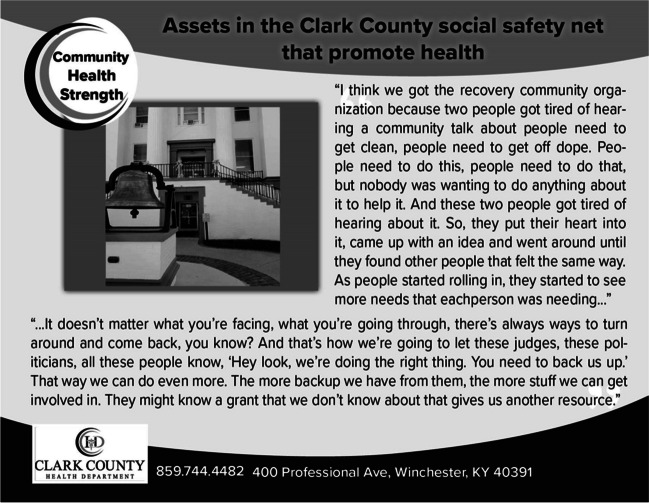
Photo and caption on postcard illustrating community health facilitator

Notably, partnerships between substance use-related nonprofits and the criminal legal system have driven a shift from a punitive to a rehabilitative approach. An employee from a recovery non-profit highlighted their strong partnership with the sheriff’s department, “what a strength they were in the community, how they’re a strong partner of (nonprofit)… They’re like family to us.” Additional community strengths were observed in partnerships between local hospitals, community service providers, courts, law enforcement, the local health department, and nonprofit organizations involved with sober living facilities and MOUD clinics. See Figs. 3 and 4 for visual representations.

#### Linking Facilitators and Barriers: the Cycle of Public Unresponsiveness to Local Calls for Behavioral Health Investments

The lack of robust public sector support emerged as a major barrier to addressing community behavioral health, prompting reliance on individual volunteers and grassroots efforts to devise and implement solutions. Many of these nonprofit and grassroots-led organizations were then identified as significant strengths, bolstered by the partnerships they formed throughout the community. The importance of these community partnerships appears to be a direct and natural response to the lack of structured behavioral health services provided by the public sector. In attempting to tackle large, systemic behavioral health issues, the decentralized nature of individual and grassroots-driven efforts compels reliance on community partnerships to bridge the gaps among fragmented services.

However, co-researchers highlighted issues associated with relying on grassroots efforts and the partnerships needed to fortify them. One explained the sheer difficulty of starting initiatives and noted that passion is not always sufficient to implement solutions: “Assets that the community does have exist because it had those champions that were willing to take the long view and to ride it out. But that doesn’t happen in every case.” They also stressed the fragility of depending on partnerships, particularly in the face of changes in relationships, funding, policy, or other social contexts. Discussing their concern regarding the continuation of a jail diversion program given an anticipated policy change, one co-researcher said, “What does that look like for that response program? With assisting those individuals?… It kind of puts a wrench in our interventions that we have in place for our community along with drug court. If they fail drug court, they’re not gonna go to where we have resources.” Another highlighted instability in a system reliant on soft-money funding for nonprofit organizations: “since they are a nonprofit and they depend on funding from the community, they’re limited on what they can and can’t do depending on how much funds they get. They always have a very tight budget.” A perceived gap existed between the work of nonprofit organizations in behavioral health and the public and philanthropic mechanisms funding this work.

With the resultant mosaic of services defended by fragile inter-organizational networks, a participant noted, “We need someone from the health department, from the recovery nonprofit, from the homeless coalition, get everybody to sit down with the city commissioners, the people with funding.” Despite frustration with the public sector, co-researchers continued to call attention to the persistent need for governmental involvement and support. Another participant noted, “We’re gonna let these judges, these politicians, all these people know, ‘Hey look, we’re doing the right thing. You need to back us up.’” Despite strong community organizations connected through partnerships, individuals involved in community-driven efforts felt the need to showcase success to secure government support. The “do it ourselves” mentality exists alongside acknowledgment of the importance of governmental support, creating a nuanced interplay where grassroots efforts led by individuals who “step up” still seek government endorsement. Another co-researcher described the complexity, saying, “It’s a complex problem that is multidimensional and the citizens have to do their piece. The government has to do their piece, the nonprofits and other organizations need to do their piece. And I guess the challenge is bringing all that together in a useful way that makes things better.” Co-researchers noted that interventions to address the behavioral health crisis should not be confined to a singular sector but instead employ a more interdisciplinary approach involving collaboration across sectors. Specifically highlighted was the need for more government support in the form of funding, exhibited by a co-researcher who stated, “It costs money to care. I mean, it takes money to actually put care into action.”

**Figure 5:**
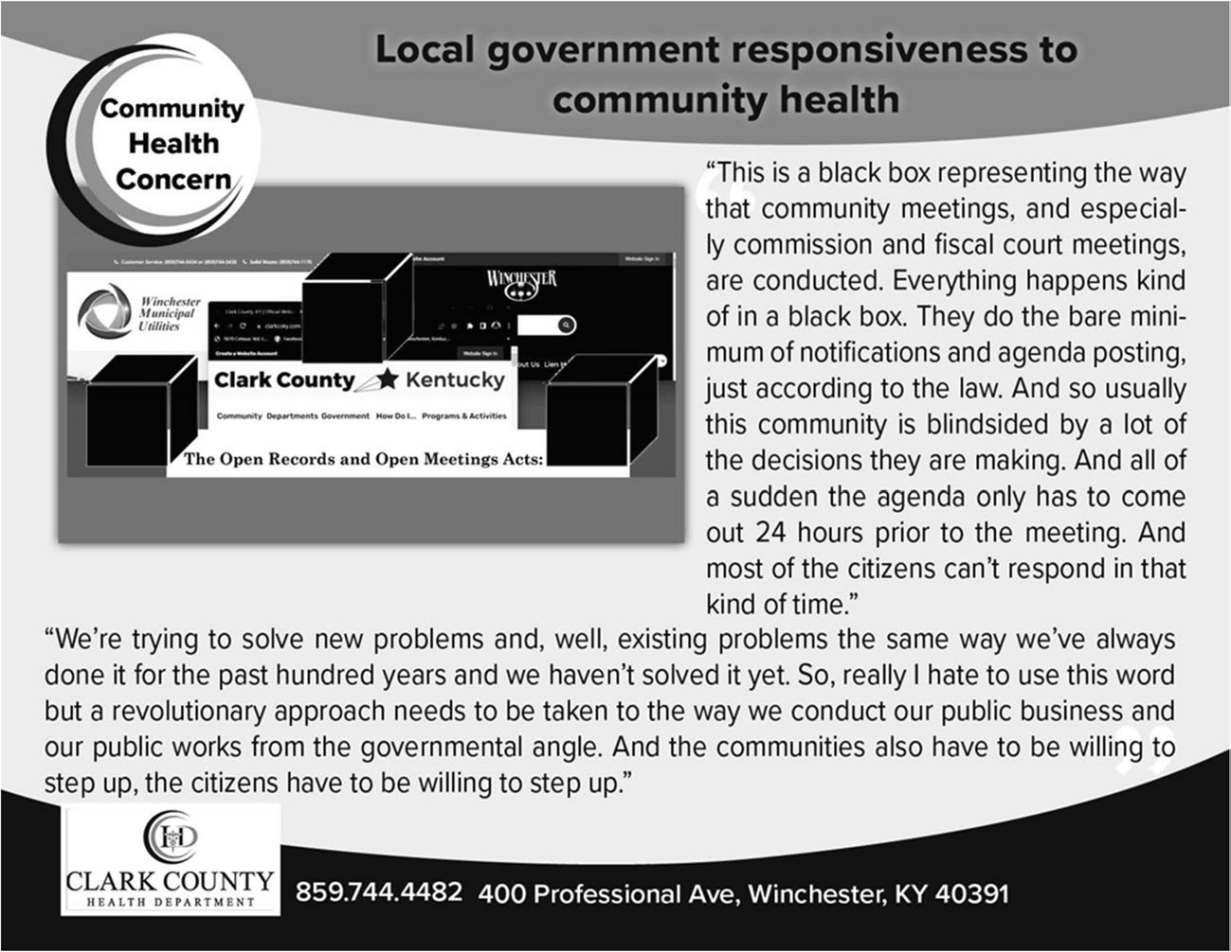
Photo and caption on postcard illustrating community health barrier

As a result, co-researchers advocated for a reassessment of behavioral health governance and the division of cross-sector responsibilities. Several suggested enhancing collaboration between the community and public sector: “I would love to experience something to know the ins and outs of our local government so then your grassroots projects could be better informed.” As illustrated in Figure 5, co-researchers emphasized the importance of cohesive action by both community and government: “A revolutionary approach needs to be taken to the way we conduct our public works from the governmental angle. And the communities also have to be willing to step up, the citizens have to be willing to step up.” By restructuring the dynamics of responsibility, addressing gaps across sectors, and matching community-identified needs to municipal resources, a more coherent and responsive system can be developed to address the multifaceted challenges of the behavioral health crisis.

Although comprehensive analysis of exhibit evaluation data is beyond the scope of this paper, it is noteworthy that these data from Photovoice co-researchers and community health forum attendees indicated that the project itself strengthened the connection between local government and community-defined behavioral health needs. Upon reflection, the second author noted that Photovoice increased non-health department engagement and showcased community ownership of the CHA process. All participants strongly agreed or somewhat agreed that the Photovoice project helped them better understand community health facilitators and barriers in Clark County, made them think more critically about how and why they exist, felt highly involved in the topic development and discussion process, and felt empowered to take action to improve community health. In post-project evaluations, a co-researcher remarked, “I think the photos showed proof of the strengths and concerns. It wasn’t just somebody talking about them. The citizens actually saw them.” Their photographic documentation became a testament to co-researchers’ concerns and advocacy. Audience members, viewing behavioral health issues through the lens of Photovoice teams, expressed transformed perceptions. One attendee stated, “It was an eye-opener for us with the struggles of drugs and alcohol in our area.” The calls to action shared at the end of each Photovoice group presentation exemplify the potential of this method to reconcile disconnects between citizen priorities and local government investments: raising awareness about the extent of homelessness intersecting with behavioral health challenges, continuing education and community collaboration to address SUD and serious mental illness, promoting the use of diversion as the primary intervention by the court system rather than incarceration, and working with local government officials to research the development of affordable housing options.

## Discussion

Although the determinants of behavioral health have been studied, few prior studies use a bottom-up community-engaged framework empowering community perceptions. Employing Photovoice, this study explores perspectives on facilitators and barriers to community behavioral health in the Clark County, KY, CHA. The results enhance our understanding of systemic assets and issues shaping the behavioral health landscape, contributing to our limited knowledge of how communities perceive links between social determinants and behavioral health. Additionally, it offers a case study on using Photovoice in CHA efforts addressing behavioral health.

Findings raise concerns about the division of responsibility in behavioral health governance. Notably, there was a call for attention to the inadequacy of public infrastructure in providing sufficient care, resources, and treatment. Consequently, a grassroots sentiment has emerged: if the public sector is unwilling or unable to address the gaps in the behavioral health continuum, then passionate individuals and impacted communities take matters into their own hands. Connections between facilitators and barriers highlight the fragility of a behavioral health system dependent on individual efforts and facing logistical challenges, unstable funding, and difficulties amid social or political changes. Community members find the division of responsibility for providing behavioral health infrastructure inadequate, asserting that the formation of individual agencies manifests as a response to insufficient or apathetic structural resources in the public sector. However, frustrations with the local government coexist with a focus on gaining governmental support. In the final member-check session, co-researchers mirrored these findings with a discussion of recent financing insufficiencies in grassroots agencies, leading the health department to absorb some behavioral health efforts previously provided by a recovery community nonprofit, and perpetuating a persistent state of precarity among services. As a result, co-researchers identified a targeted exploration of how to reformulate grassroots behavioral health efforts as a major area of future research. Emphasizing community perspectives, these findings call for greater multi-sector collaboration and coordination, bridging gaps not only between individual grassroots-led efforts, but also across the community-public-nonprofit-private continuum.

These community-based recommendations for enhanced multi-sector collaboration align with literature advocating for more integrated public health approaches to behavioral health.^65^ Behavioral health disorders are intricate conditions that interact with multiple systems—public health, healthcare, social services, government, education, and legal systems.^65^ Yet, agencies often stay siloed, failing to fully utilize each sector’s strengths to address common issues and achieve shared goals. The functional chasm that challenges multi-system crosstalk perpetuates the fragmented delivery of behavioral health services that remain the status quo, ultimately placing the burden of responsibility on impacted communities.^66,67^ While there are increasing calls for collaboration toward mutual progress, the public, private, and non-profit sectors across various disciplines continue to struggle to define where those potential overlaps may be. Identified barriers to cross-sector integration in the behavioral health continuum include the lack of accountability monitoring mechanisms,^67^ less-developed quality measurement infrastructure compared to general healthcare, stigma and discrimination disempowering behavioral health patients,^68,69^ disproportionate focus on individual treatment over social determinants, communication difficulties, and a lack of community-level data.^65^

This case study demonstrates the utility of Photovoice in addressing these barriers and its potential to construct a shared illustrative narrative from contextualized perspectives of the social determinants of behavioral health. Photographs and captions not only enhanced conventional metrics for the CCHD CHA but also provided a visual summary of community perspectives. The method fostered community engagement, empowering participants as experts to highlight important topics and frame their evaluation of the behavioral health landscape covering both individual treatment and perceived social determinants. Notably, one participant felt empowered to take action, approaching the local school board to initiate an Equity Coalition addressing racial disparities in education. Subsequent community health forums also provided opportunities for enhanced communication and collaboration. Continued impacts involved presenting the project to the Winchester Comprehensive Plan committee and engaging community members to address transportation issues. The project also continues to inform the work of the CCHD CHIP. Impacts beyond Clark County include using the project to educate aspiring public health professionals through an accredited College of Public Health Bachelor of Public Health capstone course and sparking interest from other health departments seeking to incorporate Photovoice in their behavioral health CHA/CHIP activities.

Also of notable consideration is the use of Photovoice as a quality improvement methodology. There is an emerging literature on the benefits of participatory visual techniques for quality improvement, although existing case studies remain limited and focus solely on healthcare quality rather than considerations of community health.^70–72^ Photos and captions serve as both an initial needs assessment and longitudinal monitoring tool by providing an illustrative record of community priorities over time for tracking CHIP efforts.^73^ Photovoice offers a strategy for holding public agencies and policymakers accountable for quality improvement by documenting progress or lack thereof.

The integration of Photovoice in CHAs should be considered in the context of a shifting paradigm in behavioral health from “sickcare” to healthcare to health.^74–76^ Although CHAs are well established for health departments, the Patient Protection and Affordable Care Act of 2010 added a requirement through provision 501(r)(3) for tax-exempt hospitals to triennially conduct a Community Health Needs Assessment (CHNA) and document a CHIP.^77^ Despite this potential to bridge healthcare, public health agencies, and community stakeholders, studies show that hospitals face challenges in community engagement and have wide variations in report quality and completion rates.^78–80^ Unsurprisingly, hospital CHNA reports tend to emphasize disease management, whereas health department CHA reports often prioritize community-based conditions that impact mental health and SUDs.^80,81^ There is increasing literature suggesting that hospital-health department partnerships through a collaborative CHNA/CHA process can help mitigate some of these pitfalls to enhance community health investment and improve CHNA/CHA quality.^78–80,82–85^ Unfortunately, numerous barriers limit collaboration, including inconsistencies in reporting timelines for the IRS, PHAB, and state departments of health as well as competing interests of profit vs. population health. With its focus on diverse perspectives in group dialogue, Photovoice may serve as a method to integrate CHA/CHIP and CHNA/CHIP by enhancing coordination, improving shared measurement systems, and merging data collection between hospitals and health departments.

The transformative shift to more strategic endeavors at the Kentucky state level provides a compelling context to reflect on the use of Photovoice in synchronizing public health initiatives. Under recent legislation amendments (902 KAR 8:160, Sect. 10), local health departments in Kentucky are mandated to conduct stakeholder-involved local needs assessments every 5 years.^86^^(p902)^ Future Photovoice research should focus on using this methodology to facilitate similar strategic planning for cross-jurisdictional and intergovernmental coordination. Wider use of Photovoice can build a comprehensive understanding of grassroots community needs to feed into higher-level decision-making, fostering a more responsive behavioral health system and a more effective balance of responsibility across sectors.

## Strengths and Limitations

The qualitative nature of this study within the specific context of a health department’s CHA process serves as both a strength and a limitation. Qualitative case studies are not generalizable to a population but instead allow for the expansion and analytical generalization of theories.^87^ This qualitative case study offers an in-depth analysis of community perspectives on behavioral health social determinants that is otherwise not achievable with quantitative methods. A methodological limitation involves potential self-selection bias introduced by the recruitment methods, as many co-researchers who participated were engaged in their community as volunteers, healthcare workers, and coalition members. Although contributing to informed discussions on community assets, findings may not generalize to all Clark County residents or other contexts. However, this limitation is mitigated by the representative diversity of the sample with multiple perspectives of behavioral health lived experience and across age, race, and education status in comparison to the Clark County population. The study was strengthened by a facilitation structure allowing for co-researcher participation in all project stages throughout data collection, analysis, presentation, evaluation, manuscript revision, and determining implications for producing change. This inclusive approach utilizing participants as co-researchers provides for greater ownership over research questions, data analysis, and action plans, an aspect frequently missing from other Photovoice projects.^40^

## Implications for Behavioral Health

This is the first case study utilizing Photovoice to enhance engagement and empowerment in CHAs addressing behavioral health. Findings contribute to the limited understanding of perceived links between behavioral health and social determinants. Specifically, this research uniquely explores the perspectives of community voices, providing a distinct lens to examine the discourse on the need for better cross-sector integration in addressing the behavioral health crisis. The inclusion of lived experience strengthens this research by offering nuanced perspectives on the current behavioral health landscape, highlighting that real-world perceptions of fragmentation align with the existing knowledge on the need for improved multi-sector collaboration. Photovoice offers a strategy to reconcile mismatches between grassroots behavioral health priorities and local government investments, ensuring public agency accountability through longer-term community monitoring and policy interventions. In public health crises with delayed legislative support like behavioral health, Photovoice aligns community need with a diverse range of contributing entities, offering a strategy for necessary comprehensive public health and policy interventions.

## Data Availability

The data that support the findings of this study are available on request from the corresponding author, SL. The data are not publicly available because they contain information that could compromise the privacy of research participants.
